# Perioperative changes in pro and anticoagulant factors in prostate cancer patients undergoing laparoscopic and robotic radical prostatectomy with different anaesthetic techniques

**DOI:** 10.1186/s13046-014-0063-z

**Published:** 2014-08-17

**Authors:** Maria Sofra, Anna Antenucci, Michele Gallucci, Chiara Mandoj, Rocco Papalia, Claudia Claroni, Ilaria Monteferrante, Giulia Torregiani, Valeria Gianaroli, Isabella Sperduti, Luigi Tomao, Ester Forastiere

**Affiliations:** 1 Department of Anaesthesiology, Regina Elena, Roma National Cancer Institute, Via Elio Chianesi 53, Roma 00144, Italy; 2 Clinical Pathology, Regina Elena, Roma National Cancer Institute, Rome, Italy; 3 Department of Urology, Regina Elena, Roma National Cancer Institute, Rome, Italy; 4 Division of Biostatistic, Regina Elena, Roma National Cancer Institute, Rome, Italy

**Keywords:** TIVA-TCI anaesthesia, BAL anaesthesia, Thrombotic factors, Prostate cancer, Prostatectomy

## Abstract

**Background:**

Laparoscopic prostatectomy (LRP) may activate clotting system influencing the risk of perioperative thrombosis in patients with prostate cancer. Moreover, different anaesthetic techniques can also modify coagulant factors. Thus, the aim of this study was to investigate the effects on pro- and anti-coagulant and fibrinolytic factors of two established types of anaesthesia in patients with prostate cancer undergoing elective LRP.

**Methods:**

102 patients with primary prostate cancer, who underwent conventional LRP or robot-assisted laparoscopic prostatectomy (RALP), were studied and divided into 2 groups to receive total intravenous anesthesia with target-controlled infusion (TIVA-TCI) or balanced inhalation anaesthesia (BAL) prior to surgery. Before the induction of anaesthesia (T0), 1 hr (T1) and 24 hrs post-surgery (T2), some pro-coagulant factors, fibronolysis markers, p-selectin and haemostatic system inhibitors were evaluated.

**Results:**

Both TIVA-TCI and BAL patients showed a marked and significant increase in pro-coagulant factors and consequent reduction in haemostatic system inhibitors in the early post operative period (*p*???0.004 for each markers). Use of RALP showed a significant increase in prothrombotic markers as compared to LRP. In TIVA patients undergoing LRP, a significant reduction of p-selectin levels between T0 and T2 (p?=?0.001) was observed as compared to BAL, suggesting a better protective effect on platelet activation of anaesthetic agents used for TIVA.

**Conclusions:**

Both anaesthetic techniques significantly seem to increase the risk of thrombosis in prostate cancer patients undergoing LRP, mainly when the robotic device was utilized, encouraging the use of a peri-operative thromboembolic prophylaxis in these patients.

## Background

Several epidemiological studies have shown that a strong correlation exists between cancer and haemostatic system [1]-[4]. The interaction between cancer and the coagulation system perturbs and stimulates pro-coagulant activity, consequently inducing a pro-thrombotic state [5] and increasing the risk of thromboembolic disease (TED) [6]. Interestingly in cancer patients a systemic activation of blood coagulation has frequently been observed even in the absence of TED [2],[7].

**Figure 1:**
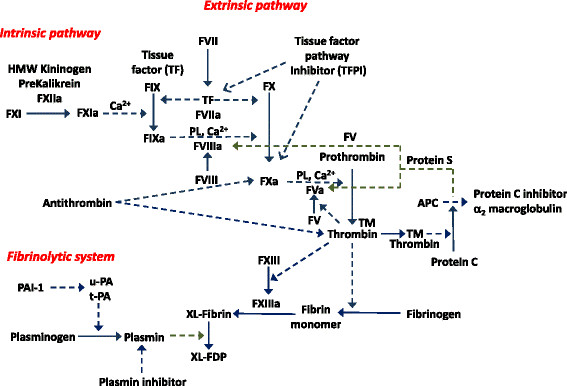
**Coagulation cascade.** The solid lines indicate a activating function, while the dashed lines a inhibitory action.

Cancer cells can activate the clotting system directly, thereby generating thrombin, or indirectly by stimulating mononuclear cells, platelets and endothelial cells to synthesize and express a variety of procoagulants [8]. The consequent formation of a fibrin matrix appears to promote tumor growth by favoring neoangiogenesis and shielding tumor cells against attack from immunocompetent cells [5]. Thrombin also works as a potent promoter of cancer growth and spread via an increase in tumor cell adhesion [9]. Some biomarkers have been specifically investigated for their capacity to predict TED during the course of cancer disease. Associations between elevated levels and future TED have been found for D-Dimer, prothrombin fragment 1?+?2 (F1?+?2), thrombin-antithrombin complexes (TAT), plasminogen activator inhibitor type 1 (PAI-1), clotting factor VIII (FVIII) and soluble P-selectin [10]. These markers, not sufficiently validated in patients undergoing different intraoperative anaesthetic regimens, reflect different steps of the coagulation cascade (Figure 1). In particular, F1?+?2 is released when activated factor X cleaves prothrombin into active thrombin and the fragment formation is a key event in the coagulation cascade. The formation of TAT complexes represents an indirect measure for the activation of the coagulatory system, because is the first amount of thrombin that binds to antithrombin (AT). Elevated FVIII levels are a well-established risk factor for first manifestation and for recurrence of TED. PAI-1 is a potent inhibitor of the fibrinolytic system while d-dimer is a stable end product of fibrin degradation and is elevated by enhanced fibrin formation and fibrinolysis [10]-[12]. P-selectin, a member of cell adhesion molecules, is released from the ?-granules of activated platelets and from Weibel-Palade bodies of endothelial cells. P-selectin plays a crucial role in thrombogenesis and induces a prothrombotic state by the adhesion of platelets and leukocytes to cancer cells. Levels of soluble P-selectin are elevated in patients with acute TED [13].

Surgical tissue trauma also leads to an increased risk of TED [14] even though the incidence of TED is closely related to the organ involved. The tumor sites most at risk of developing TED seem to be the pancreas, brain, and stomach [14]. In patients with advanced prostate cancers, the incidence of TED is controversial, ranging from 0.5% to 40% in the first month after surgery [3],[15]-[17]. The increased risk of TED in prostate cancer patients undergoing radical prostatectomy recommends administering a pharmacologic anti-thrombotic prophylaxis [18]-[22], though the latter may cause an increase in intra-operative bleeding [23],[24] .

To date, factors influencing the risk of perioperative thrombosis in patients undergoing prostate cancer surgery have not been identified yet. At present, we do not know whether, in addition to the risk factors already known, the use of different techniques of anesthesia may increase the risk of thrombosis in cancer patients undergoing surgery. Therefore, the main aim of this prospective study was to investigate changes in the markers most sensitive to detecting activation of the haemostatic system in patients with prostate cancer undergoing elective laparoscopic prostatectomy with two different intra-operative anaesthetic regimens, target-controlled infusion (TIVA-TCI) and balanced inhalation anaesthesia (BAL). A secondary aim was to evaluate whether using a robot device in the laparoscopic prostatectomy influences the effect of different anesthetic techniques applied.

## Methods

### Patient population

**Figure 2:**
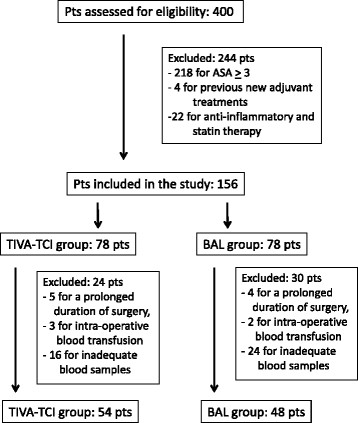
Design of the study: patient selection.

Between October 2009 and June 2012, 400 consecutive patients with primary prostate cancer, undergoing general anaesthesia and conventional laparoscopic radical prostatectomy (LRP) or robot-assisted laparoscopic prostatectomy (RALP), were considered eligible for the study (Figure 2). This study was approved by the Ethics Committee of the Regina Elena National Cancer Institute, Rome (Prot.CE/550), and a written informed patient consent was obtained from all participants. Protocol was registered in *Clinical trials.gov* (NCT01998685). The inclusion criteria for the study were a newly diagnosed cancer of the prostate with histological Gleason score evaluation. Exclusion criteria included: (a) ASA >2, (b) metabolic equivalent task?<?4, (c) BMI?>?30, (d) no pre-operative pharmacological thromboprophylaxis and/or anti-coagulant therapy, (e) history of abnormal bleeding, or abnormal coagulant factors, (f) sepsis within the last 2 weeks, (g) previous new adjuvant treatments (chemo, hormone, and radiotherapy), (h) non-steroid, anti-inflammatory and statin drugs for at least 2 wks before surgery, (i) venous or arterial thromboembolism within the last 3 months, peripheral venous disease, (l) neurological disease with extremity paresis, (m) chronic liver disease, (n) pre-operative haemoglobin concentration?<?9 mg dl^?1^, (o) prolonged duration of surgery (>3 hrs); (p) peri-operative blood transfusion, (q) inadequate material for laboratory testing. One exclusion criterion sufficed exclusion.

Out of the 400 patients with primary prostate cancer who underwent laparoscopic prostatectomy, 244 were excluded from the study for the following reasons: 218 for ASA???3, 4 for previous new adjuvant treatments, 22 for anti-inflammatory and statin therapy before surgery. Thus, 156 patients with primary prostate cancer constituted the patient population of this randomized study and were alternatively divided into 2 groups to receive TIVA-TCI or BAL anaesthesia prior to surgery. Then, a further 54 patients were excluded: 9 for a prolonged duration of surgery, 5 for intra-operative blood transfusion and 40 for inadequate blood samples. Finally, 102 patients with primary prostate cancer comprised the patient population of the study: 54 received TIVA-TCI and 48 BAL anesthesia prior to surgery.

All patients with high-risk prostate cancer (according to Guidelines on Prostate Cancer of European Association of Urology, 2012) underwent LRP with extended pelvic lymph node dissection. Patients with intermediate risk underwent LRP or RALP .

### Anesthetic protocol

The patients did not receive premedication. In the TIVA-TCI group, anaesthesia was induced with propofol (Diprivan^TM^, ASTRA-Zeneca, Milano, Italy) 6 ?g ml^?1^ and remifentanyl (Ultiva^TM^, GlaxoSmith-Kline AB, Verona, Italy) 0.4-1 ?g kg^?1^ min, simultaneously administered using two separate modules of a continuous computer-assisted TCI system. Anaesthesia was maintained with propofol 4 ?g ml^?1^ and remifentanil 0.25 ?g Kg^?1^ min. This infusion was modified by 0.05 ?g kg^?1^ min steps according to analgesic needs. In the BAL group, anaesthesia was induced with midazolam (Hameln pharmaceuticals Gmbh, Hameln, Germany) 0.1 mg kg^?1^ and fentanyl (Fentanest^TM^, Pftzer, Latina, Italy) 1.5 ?g kg^?1^ Anaesthesia was maintained with sevoflurane (Sevorane^TM^, Abbott, Latina, Italy) 2.0% , oxygen 40% and air 70% with positive pressure ventilation in a circle system, in order to achieve normocapnia.

In both groups, cisatracurium besylate (Nimbex^TM^, Glaxo Smith Kline) 0.1-0.5 mg kg^?1^ was given to facilitate orotracheal intubation with a cuffed tube, followed by the continuous application of 0.06-0.12 mg kg^?1^ h^?1^ via infusion pumps. Pneumoperitoneum was created by intraperitoneal insufflation of CO_2_ with an insufflation pressure of 13¿15 mmHg and patient in the supine position. Patients were then placed in the steep Trendelenburg position (30° from horizontal). Intraperitoneal pressure was maintained at 15 mmHg during the induced pneumoperitoneum. A routine anaesthesia monitoring was performed on all patients (Table 1).

**Table 1:** Clinical characteristics and peri-operative data of patients with prostate cancer who underwent surgery with TIVA-TCI or BAL anaesthesia

	**TIVA-TCI (n. 54)**	**BAL (n. 48)**	**P**
**Clinical data**			
?? **Age (yrs)**	60.66 (5.91)	62.16 (6.23)	0.31
?? **Venous thromboembolism risk**			
?? Highest risk	54 (100%)	48 (100%)	1
?? Prostate cancer risk*			
?? Intermediate-risk	26 (48.1%)	30 (62.5%)	
?? High-risk	28 (51.8%)	18 (37.5%)	0.17
?? **ASA, n (%):**			
?? I	4 (7.4%)	6 (12.5%)	
?? II	50 (92.6%)	42 (87.5%)	0.39
?? **Histological grade of cancer**			
?? G2 (Gleason 5¿6)	15 (27.8%)	14 (29.2)	
?? G3 (Gleason 7¿10)	39 (72.2%)	34 (70.8%)	0.88
?? **pT, n (%)**			
?? 2	30 (55.6%)	32 (66.7%)	0.25
?? 3	24 (44.4%)	16 (33.3%)	
?? **pN, n (%) #**			
?? 0	17 (85.0%)	24 (96.0%)	0.20
?? 1	3 (15.0%)	1 (4.0%)	
**Peri-operative data**			
?? **Type of surgery**			
?? LRP	36 (66.7%)	34 (70.8%)	0.65
?? RALP	18 (33.3%)	14 (29.2%)	
?? **Time of anaesthesia (min)**	107.5 (16.8)	101.4 (26.2)	0.26
?? **Blood loss (ml)**	123.3 (131.1)	121.4 (110.6)	0.81
?? **Total amount of crystalloid received (ml)**	468.5 (110.21)	496.8 (198.5)	0.27
?? **Intra-operative body temperature**	36.2 (0.3)	36.1 (0.2)	0.83
?? **Intra-operative MAP (mmHg)**	104.6 (10.5)	106.2 (10.2)	0.61
?? **Intra-operative SpO2 (%)**	96.7 (0.9)	97.8 (1.8)	0.75
?? **Arterial lactate level (mmol/l)**			
?? 1 h post-surgery	0.7 (0.2)	0.6 (0.4)	0.32
?? 24 h post-sugery	1.7 (0.2)	1.8 (0.2)	0.82
?? **Intra-operative BE (mmol/l)**	0.3 (0.4)	0.4 (0.4)	0.62
?? **Intra-operative PaO2 (mmHg)**	219.4 (11.2)	216.5 (16.8)	0.72

Values are expressed in absolute values or mean (SD).

*Abbreviations*: *TIVA-TCI* total intravenous anaesthesia with target-controlled infusion, *BAL* balanced inhalation anaesthesia, *LRP* conventional laparoscopic radical prostatectomy, *RALP* robot-assisted laparoscopic prostatectomy.

*According to Guidelines on Prostate Cancer, European Association of Urology, 2012.

#Lymph node dissection was made in 45 out of 102 pts.

During anaesthesia all patients received warm venous infusion of saline solution (0.9% NaCl) 3 ml Kg ^?1^?h^?1^ and thermal mattresses. Systolic arterial pressure was maintained at 100 mm Hg or 70% of the preoperative value. Hypotension was treated with crystalloid fluid infusion or intravenous boluses of ephedrine.

After surgery the residual neuromuscular blockade was reversed with a mixture of atropine (Galenica Senese, Siena, Italy) 1.5 mg and neostigmine (Intrastigmina^TM^, Lusofarmaco, Milano, Italy) 2.5 mg. Anaesthetic agents were switched off, and 100% O_2_ was given with 8 l min fresh gas flow for 1 min. In addition, a forced-air warming blanket was used post-surgery (Equator Covective Warming ^TM^, Smith Medical Italia, Milano, Italy).

After tracheal extubation all patients received ketoralac trometamina (Toradol, Recordati, Milano, Italy) 30 mg, ranitidine (Ranidil^TM^, Menarini, Firenze, Italy) 50 mg and morphine (Recordati) 2 mg in bolus and then by a controlled analgesia device (Deltec^TM^, Smiths Medical ASD, St Paul, MN).

### Clinical parameters

The risk of venous thromboembolism was evaluated according to the model proposed by Caprini et al. [25] and Bergqvist et al. [26]. Patients were divided into 4 different levels of risk: low (score 0¿1), moderate (score 2), high (score 3¿4), highest (score >4). The following clinical parameters were also evaluated: (a) global assessment of anesthetic risk (ASA), (b) grading of prostate cancer (Gleason score), (c) pathological tumor-node-metastasis stage, (d) time of surgery, (e) quantity and type of liquids administered, (f) blood loss, (g) peri-operative complications such as hypertension, hyperglycemia, hypothermia, infections and pain (evaluated by a 6-point verbal rating scale: 0: no pain to 5: most severe pain imaginable).

In all patients, the presence of venous thrombosis by clinical observation, venous and pelvic ultrasound were evaluated in the peri-operative period and on days 8 and 21 after surgery.

### Prophylaxis anti-thrombosis

Since in most of our patients changes in pro- and anti-coagulant and fibrinolytic markers were observed in the peri-operative period, an anti-thrombotic prophylaxis was made 24 hrs post surgery, for 4 weeks, by using Enoxaparina (Clexane^TM^, Sanofi-Aventis, Milano) 4000 UI/die .

### Prothrombotic markers

Before the induction of anaesthesia (T0), 1 hr post-surgery (T1) and 24 hrs post-surgery (T2), the following factors were evaluated: (a) procoagulant markers: fibrinogen, TAT, F1?+?2 and FVIII; (b) fibrinolysis markers: PAI-1, D-dimer; (c) platelet-aggregating properties: p-selectin; (d) hemostatic system inhibitors: AT, protein C (PC) and protein S (PS) activity.

Blood samples were collected in tubes without additives containing 3.2% sodium citrate (Vacutainer, Becton-Dickinson, Franklin Lakes, NJ USA). Samples were centrifuged within 1 h at 2500 g for 20 min, to obtain platelet-poor plasma. The plasmas were immediately tested. Moreover, plasma and serum samples were separated and stored in multiple aliquots at ?80°C for subsequent testing. All coagulation parameters (PT, aPTT, fibrinogen, AT, D-dimer, PC, PS, FVIII) were assayed by clotting, chromogenic and immunological methods on fully-automated ACL TOP analyzer using HemosIL® commercial kits (Instrumentation Laboratory Company, Bedford, MA USA). Abnormal values were defined by the clinical laboratory or manufacturer¿s assay. Plasma levels of TAT and F1?+?2 were measured by enzyme-linked immunosorbent assay Enzygnost® TAT micro and Enzygnost® F1?+?2 mono kits, respectively (Siemens Healthcare Diagnostics Inc, NY USA), according to the manufacturer¿s instructions. Both assays employ the quantitative sandwich enzyme immunoassay technique. All samples showing values above the standard curve were re-tested with appropriate dilutions. Plasma levels of PAI-1 were measured with the enzyme-linked immunosorbent assay Asserachrom® kit (Diagnostica Stago, Asnieres, France), according to the manufacturer¿s instructions. Plasma p-selectina levels were determined by Human sP-Selectin enzyme immunoassay (R&D Systems, Inc Minneapolis, MN USA), according to the manufacturer¿s instructions, employing the quantitative sandwich enzyme immunoassay technique.

### Statistical analysis

Data were analyzed with Statistical Package for the Social Sciences (SPSS) 14.0 software. Continuous and categorical variables were expressed as the mean?±?standard deviation or standard error and as frequency values and proportions, respectively. Pearson¿s chi-square test was used to assess possible differences in dichotomous variables between the various groups examined. The means of normally distributed data were compared with the Student¿s *t-*test. In other cases, the groups were compared with the Mann-Whitney¿s *U* test. P values of the tests were adjusted using the Bonferroni method. Paired samples were analyzed by *t*-test and Wilcoxon Signed Ranks Test. Multiple linear regression was used in order to test the effect of anaesthesia, surgery and clinical characteristics of patients on changes of prothrombotic markers 24 h post-surgery (T2 time). A p-value of <0.05 was considered statistically significant.

## Results

### Clinical characteristics of the patients

The clinical characteristics of the patients enrolled in the study are reported in Tables 1 and 2. No significant differences were observed regarding age between TIVA-TCI and BAL patients.

**Table 2:** Clinical characteristics and peri-operative data of patients with prostate cancer, divided in 4 subgroups according type of anesthesia and surgery

	**TIVA-TCI LRP (n. 36 pts)**	**TIVA-TCI RALP (n. 18 pts)**	**BAL LRP (n. 34 pts)**	**BAL RALP (n. 14 pts)**	**P**
**Clinical data**					
?? **Age (yrs)**	61.4 (5.7)	59.5 (6.7)	63.2 (5.8)	60.1 (7.7)	0.25
?? **ASA, n (%):**					
?? I	3 (8.3%)	1 (5.6%)	5 (14.7%)	1 (7.1%)	0.68
?? II	33 (91.7%)	17 (94.4%)	29 (85.3%)	13 (92.9%)	
?? **Histological grade of cancer**					
?? G2 (Gleason 5¿6)	9 (25.0%)	6 (33.3%)	10 (29.4)	4 (28.6)	0.93
?? G3 (Gleason 7¿10)	27 (75.0%)	12 (66.7%)	24 (70.6%)	10 (71.4%)	
?? **pT, n (%)**					
?? 2	12 (33.3%)	18 (100%)	18 (52.9%)	14 (100%)	0.001
?? 3	24 (66.7%)	0	16 (47.1%)	0	
?? pN, n (%)*					
?? 0	11 (84.6%)	6 (85.7%)	14 (93.3%)	10 (100%)	0.57
?? 1	2 (15.4%)	1 (14.3%)	1 (6.7%)	0	
**Perioperative data**					
?? **Time of anaesthesia (min)**	104.0 (21.3)	109.7 (24.4)	98.8 (30.2)	105.2 (24.8)	0.32
?? **Blood loss (ml)**	119.2 (140.3)	128.3 (150.1)	118.2 (121.4)	125.2 (131.5)	0.30
**??Total amount of crystalloid received (ml)**	475.4 (100.4)	460.8 (118.4)	486.1 (166.4)	499.8 (200.2)	0.21
?? **Intra-operative body temperature**	36.2 (0.3)	36.1 (0.4)	36.1 (0.2)	36.1 (0.3)	0.87
?? **Intra-operative MAP (mmHg)**	103.8 (11.8)	105.3 (12.5)	105.4 (12.4)	106.8 (12.2)	0.54
?? **Intra-operative SpO2 (%)**	96.7 (0.9)	96.7 (0.9)	97.8 (1.8)	97.8 (1.8)	0.75
?? **Arterial lactate level (mmol/l)**					
?? 1 h post-surgery	0.7 (0.2)	0.7 (0.3)	0.6 (0.3)	0.6 (0.4)	0.81
?? 24 h post-sugery	1.8 (0.3)	1.7 (0.2)	1.7 (0.3)	1.8 (0.3)	0.77
?? **Intra-operative BE (mmol/l)**	0.3 (0.4)	0.4 (0.3)	0.3 (0.4)	0.4 (0.3)	0.78
?? **Intra-operative PaO2 (mmHg)**	220.6 (13.2)	218.8 (13.4)	214.6 (18.6)	219.5 (19.0)	0.22

Values are expressed in absolute values or mean (SD).

*Abbreviations*: *TIVA-TCI* total intravenous anaesthesia with target-controlled infusion, *BAL* balanced inhalation anaesthesia, *LRP* laparoscopic radical prostatectomy, *RALP* robot-assisted laparoscopic prostatectomy.

*Lymph node dissection was made in 45 out of 102 pts.

Thirty-two out of 102 patients (31.4%) underwent RALP and were equally distributed between the TIVA-TCI and BAL. The lymph node dissection was made in 45 out of 102 pts (44.1%).

All patients were at highest risk of venous thromboembolism, according to the model proposed by Caprini et al. [25] and Bergqvist et al. [26] (being all neoplastic and undergoing surgery); 10 of these (9.8%) had an ASA I whereas 92 (90.2%) an ASA II.

Thirty-nine patients of TIVA-TCI group (72.2%) and 34 of BAL group (70.8%) showed a high grade prostatic carcinoma (G3) with Gleason score ?7.

Patients undergoing LRP showed a locally more advanced tumor (pT3) as compared to those treated with RALP (Table 2). No significant differences were observed regarding lymph node involvement (pN). The mean duration of anesthesia was 103.8?±?26.1 min, with no differences between the TIVA-TCI and BAL groups (p?=?0.26).

During surgery a light decrease in hematocrit and hemoglobin concentration was observed in both groups, but intra-operative blood loss was similar. Also, the volume of crystalloid administered during anaesthesia was similar in both groups. Similarly, no statistical differences were observed regarding hemodynamic and respiratory parameters. None of the patients experienced adverse clinical events during their postoperative course.

In all patients no TED was observed in the post-operative period and in a 2-yr follow-up. This is probably due to the anti-thrombotic prophylaxis which was carried out for ethical reasons in all patients 24 hrs post surgery because intra-operative changes of some pro-coagulant markers were observed. Lymph node metastases were detected in only 4 out of 45 patients with lymph node dissection (8.9%): one in the TIVA-TCI group and 3 in the BAL group (p?=?0.32).

### Types of anaesthesia and prothrombotic markers

Changes of prothrombotic markers associated with the use of different techniques of anesthesia are reported in Tables 3 and 4. No statistically significant differences were observed in the baseline values of biomarkers (at T0) between TIVA-TCI and BAL groups, even when we considered the type of surgery. In both TIVA-TCI and BAL patients a significant and continuous reduction in screen clotting time PT (given as percentage) was observed during post-surgery period (T2) as compared to T0 (p?=?0.001), while aPTT was shortened at T1 and then normalised on the first postoperative day (T2).

**Table 3:** Changes of prothrombotic markers in patients with prostate cancer who underwent surgery with total intravenous anesthesia with target-controlled infusion (TIVA-TCI) before the induction of anaesthesia (T0), 1 hr post-surgery (T1) and 24 hrs post-surgery (T2)

	**T0**	**T1**	**T2**	**P**		
				**T0 vs T1**	**T1 vs T2**	**T0 vs T2**
**Screen clotting time**						
- PT (%)	93.1 (1.3)	85.6 (1.2)	82.5 (1.2)	0.001	0.21	0.001
- PTT (sec)	29.6 (0.6)	26.8 (0.7)	27.6 (0.8)	0.003	0.07	0.18
**Procoagulant markers**						
- Fibrinogen (mg/dL)	285.5 (7.1)	262.3 (6.6)	353.3 (8.8)	0.004	0.001	0.001
- TAT (ng/L)	9.1 (1.9)	22.8 (3.2)	9.7 (2.4)	0.002	0.004	0.79
- F1?+?2 (pmol/L)	210.8 (27.3)	622.1 (64.2)	364.4 (45.6)	0.001	0.001	0.007
- FVIII (%)	142.9 (8.1)	194.2 (9.3)	162.3 (5.6)	0.001	0.004	0.04
**Fibrinolysis markers**						
- PAI-1 (ng/ml)	15.2 (1.4)	21.9 (5.8)	36.1 (9.8)	0.41	0.20	0.04
- D-dimer (?g/L)	127.1 (12.8)	721.4 (170.4)	364.2 (28.3)	0.001	0.02	0.001
**Haemostatic system inhibitors**						
- AT (%)	102.1 (1.8)	90.6 (1.9)	87.4 (2.4)	0.001	0.38	0.001
- protein C (%)	109.6 (2.8)	95.4 (2.8)	87.8 (2.8)	0.004	0.03	0.001
- protein S (%)	93.8 (3.1)	84.2 (2.8)	82.4 (2.4)	0.01	0.56	0.001
**Platelet-aggregating properties**						
- p-selectin (ng/ml)	37.9 (2.0)	36.8 (2.4)	33.5 (2.6)	0.78	0.37	0.28

Values are mean (SD).

**Table 4:** Changes of prothrombotic markers in patients with prostate cancer who underwent surgery with balanced inhalation anaesthesia (BAL) before the induction of anaesthesia (T0), 1 hr post-surgery (T1) and 24 hrs post-surgery (T2)

	**T0**	**T1**	**T2**	**P**		
				**T0 vs T1**	**T1 vs T2**	**T0 vs T2**
**Screening clotting time**						
- PT (%)	91.4 (1.4)	86.8 (1.6)	81.8 (1.4)	0.007	0.02	0.001
- PTT (sec)	30.1 (0.4)	26.2 (0.7)	28.3 (0.6)	0.001	0.02	0.01
**Procoagulant markers**						
- Fibrinogen (mg/dL)	318.5 (8.6)	301.3 (10.9)	372.4 (11.2)	0.21	0.001	0.001
- TAT (ng/L)	6.2 (0.8)	19.2 (3.1)	6.7 (0.8)	0.002	0.002	0.42
- F1?+?2 (pmol/L)	182.4 (11.8)	558.1 (65.6)	266.8 (19.2)	0.001	0.001	0.001
- FVIII (%)	123.4 (4.8)	228.2 (15.8)	169.2 (6.2)	0.001	0.001	0.001
**Fibrinolysis markers**						
- PAI-1 (ng/ml)	14.1 (1.4)	21.7 (15.8)	22.6 (2.4)	0.16	0.86	0.002
- D-dimer (?g/L)	175.5 (22.6)	622.1 (175.4)	421.3 (30.6)	0.003	0.07	0.001
**Haemostatic system inhibitors**						
- AT (%)	97.8 (1.7)	92.0 (1.7)	89.1 (1.8)	0.04	0.25	0.001
- protein C (%)	105.2 (3.8)	99.3 (2.7)	88.5 (2.7)	0.18	0.03	0.001
- protein S (%)	95.6 (2.4)	91.2 (2.4)	81.8 (2.6)	0.08	0.01	0.001
**Platelet-aggregating properties**						
- p-selectin (ng/ml)	41.5 (2.7)	40.7 (2.9)	40.2 (2.8)	0.65	0.88	0.18

Values are mean (SD).

At the end of surgery (T1), both TIVA-TCI and BAL patients showed a marked and significant increase in pro-coagulant factors (TAT, F1?+?2 and FVIII) and consequent reduction in haemostatic system inhibitors (AT, PC and PS) compared to the values measured prior to surgery (*p*???0.004 for each markers). The greatest increase was observed in the values of TAT and F1?+?2 (about 3 times compared to T0), while the values of FVIII increased approximately 30%. F1?+?2 and FVIII slightly reduced at T2 but remained significantly higher than basal levels (p???0.04 for each markers). Only TAT values returned to pre-anaesthesia values. We observed a corresponding increase in anti-coagulant factors that remains significantly lower than prior to surgery (p?=?0.001).

Fibrinogen levels significantly decreased at T1 in comparison to the initial values, but rose significantly 24 hours post-surgery in both groups, showing an increase of about 20-30% as compared to T0 values (p?=?0.001).

Changes in pro-coagulant factors and haemostatic system inhibitors were similar in both TIVA-TCI and BAL patients with no significant differences between the two groups of patients. In regards to the fibrinolysis system, D-dimer concentration in TIVA-TCI group, levels increased about 6-fold at T1 compared to baseline level (p?=?0.001, Table 3), while in BAL patients it showed an increase of about 4-fold (p?=?0.001, Table 4). Both groups showed a decrease of D-dimer at T2 even if the concentration remained higher than baseline levels (p?=?0.001), with no significant differences between TIVA-TCI and BAL patients.

Levels of the PAI-1, the principal inhibitor of the fibrinolysis system, and D-dimer remained constant between T0 and T1 but significantly increased at T2 in both groups.

Grading of prostate cancer evaluated by Gleason score and pathological tumor stages showed no significant effects on changes in prothrombotic markers observed both in the TIVA and BAL groups. Similarly, it was observed for all other clinical parameters analyzed.

### Surgery and prothrombotic markers

**Figure 3:**
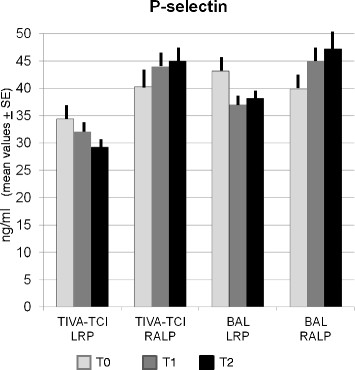
**Changes of p-selectin levels between T0 (before the induction of anaesthesia) and T2 (24 hrs post-surgery) in patients undergoing conventional laparoscopic radical prostatectomy (LRP) or robot-assisted laparoscopic prostatectomy (RALP).** TIVA-TCI patients undergoing LRP showed a significant reduction in p-selectin levels between T0 and T2 (p?=?0.001) while no changes were observed in the BAL group. In contrast, a significant increase of p-selectin value was observed 24 hours after surgery (T2) in patients undergoing RALP, regardless of the type of anaesthesia.

Multivariate analysis demonstrated that only p-selectin was significantly correlated to the type of anesthesia and surgery (p?=?0.01). It is very important to note that the TIVA-TCI patients undergoing LRP showed a significant reduction in p-selectin levels between T0 and T2 (p?=?0.001) while no changes were observed in the BAL group that did not use the robotic device (Figure 3). In contrast, a significant increase of p-selectin value was observed in patients undergoing RALP, regardless of the type of anesthesia, both 1 and 24 hours after surgery.

Patients undergoing RALP showed also 24 hrs after surgery (T2), at univariate analysis, a greater reduction of PS, an inhibitor of haemostatic system, as compared to patients undergoing LRP (p?=?0.02) independent of the type of anaesthesia applied.

## Discussion

Results of our study have demonstrated that both anaesthetic techniques seem to increase the risk of TED in prostate cancer patients undergoing LRP, mainly when the robot device was utilized, suggesting, therefore, the utility of a peri-operative thromboembolic prophylaxis. In fact, both TIVA-TCI and BAL patients showed a marked and significant increase in pro-coagulant factors and consequent reduction in haemostatic system inhibitors in the early post operative period (*p*???0.004 for each markers). However, this effect could be linked also to surgical stress, although the latter seems to have an independent effect only for p-selectin, as demonstrated by multivariate analysis. Moreover, the significant reduction of p-selectin levels between T0 and T2 (p?=?0.001) observed in TIVA patients undergoing LRP, although this group of patients was composed mainly of patients at high-risk prostate cancer (as reported in Table 1), demonstrated that general anaesthetic agents used for TIVA have a better protective effect on the platelet activation in this subgroup of patients.

The evaluation of markers detecting activation of the hemostatic system represents a more sensitive way to assess the risk of thromboembolism as compared to the clinical assessment of TED. In our study, the activation of haemostatic system associated with thromboembolic risk was estimated by measuring levels of thrombin activation markers. TAT, PF1?+?2 and FVIII increased in the immediate post operative period and gradually returned to near baseline levels. The peri-operative activation of coagulation also caused an increased of peri-operative PAI-1 levels, a potent inhibitor of fibrinolysis. The activation state persists during surgery and is independent of the anaesthetic agents used. These results confirm previous studies performed on patients undergoing major abdominal surgery for colon-rectal cancer [27], hepatic cancer resection [28], pneumonectomy for lung cancer [29].

No studies had previously examined whether different intra-operative anaesthetic regimens (TIVA-TCI *vs.* BAL) could cause different intra-operative profiles of highly sensitive and specific coagulation and fibrinolysis markers in prostate cancer patients undergoing a highly standardized type of surgery (LRP or RALP). In this context, the results of our study seem to provide useful information in reducing the peri-operative trombo-embolic risk and improving the prognosis of cancer patients undergoing LRP and RALP.

Even though cancer patients who undergo surgery are targeted for thromboprophylaxis, widespread use of prophylaxis could determine the risk of intra-operative bleeding [23],[24] and a detrimental effect rather than a benefit. This problem is evident in prostate cancer patients undergoing surgery, especially in view of the increasingly frequent use of the robotic technique that has resulted in a significant reduction of surgical complications [30],[31]. Although the American and European guidelines recommend prophylaxis in patients with prostate cancer [18]-[22], its use is currently widely debated given the different incidence of TED observed by several authors. A multicentric analysis of a number of institutions from both Europe and the United States showed a very low incidence of TED (about 0.5%) [32]. A similar incidence (0.9%) was reported from the California Cancer Registry [4]. Conversely, Osborne et al. [14] consider patients with prostate cancer at intermediate risk of TED similar to patients with uterine, rectal, colon and liver cancer.

Prostatectomy significantly increases the incidence of TED up to 2.9% and 3.9%, as reported by Hu JC et al. [17], irrespective of the surgical approach. Tewari et al. [33] in a recent meta-analysis on 400 original research articles on surgical treatment for prostate cancer and its complications reported that the rate of deep vein thrombosis was significantly lowest for RALP (0.3%), intermediate for LRP (0.5%) and highest for open surgery (1.0%). More recently, Van Hemelrijck et al. [16] analysed thromboembolic events following prostatectomy in about 45.000 men collected in the Prostate Cancer Database Sweden. Risk of venous thromboembolism and pulmonary embolism occurred especially in the first 2 months after surgery with the highest risk in patients undergoing open or laparoscopic surgery with pelvic lymph node dissection while laparoscopic procedures without lymph node dissection were at lowest risk. Unfortunately, in this study authors did not created separate categories for LRP and RALP as the majority of laparoscopic surgery was performed with robotic assistance. In our case series, dissection of pelvic lymph node was not an independent risk factor for TED because no significant differences were demonstrated in the values of the markers analyzed among the various subgroups of patients studied. Moreover, it should be noted that in previous studies only the clinical incidence of venous thromboembolism was measured, but not the changes of coagulation factors. In other studies many biomarkers were specifically checked for their capacity to predict venous thromboembolism during the course of cancer disease [10], but changes in these markers due to different types of surgery, such as LRP or RALP, were not evaluated. Our results are even more surprising when we consider that the anesthetic drugs used both in TIVA-TCI and BAL, in particular propofol [34] and sevoflurane [35], act by inhibiting the platelet aggregation, although with different mechanisms.

Patients underwent RALP, compared to LRP group, showed a greater reduction of inhibitors of haemostatic system, such as protein S, and the increase of p-selectin, a cell adhesion molecule on the surface of activated endothelial cells and activated platelets [13]. Data present in the literature regarding the different risk of thrombosis in patients submitted to LRP or RALP are very few. In a recent study Saily et al. [36] observed that RALP activates coagulation, and thromboprophylaxis for high-risk patients even after minimally invasive surgery may be beneficial. In particular, patients undergoing RALP showed postoperatively increased levels of fibrinogen, factor VIII, d-dimer associated to a thrombocytosis, reflecting a coagulation activity. The greater risk of thrombosis with the RALP could be also related to the surgical stress that leads RALP to a major release of inflammatory mediators [37] or a greater oxidative stress induced by ischemia¿reperfusion [38], determining the endothelial dysfunction and hypercoagulability [27]. This hypothesis is outlined by the fact that no differences were observed in other factors that may cause an activation of the haemostatic system in the peri-operative period such as anemia, hypoxia, hypothermia, hemodilution, hypotension, peritoneal insufflation, and Trendelenburg position [39],[40]. We do not know whether changes in pro-coagulant factors may determine the occurrence of thrombotic complications since an anti-thrombotic prophylaxis was administered for ethical reasons 24 hrs after surgery.

Our results suggest the use of a prophylaxis in all patients undergoing laparoscopic prostatectomy, in particular RALP, regardless of the type of anesthesia. Prophylaxis could not be required only in patients undergoing LRP with TIVA-TCI anaesthesia since a significant reduction in p-selectin levels between T0 and T2 (p?=?0.001) was observed in this subgroup of patients. On the contrary, p-selectin did not change in patients undergoing LRP with BAL. Thus, the results we obtained suggest a greater inhibition effect of propofol, as compared to sevofluorane, on platelet aggregation p-selectin mediated. The different effect of propofol and sevofluorane on p-selectin levels observed in our study is in agreement with previous observations reporting that sevofluorane inhibits human platelet aggregation induced by weak antagonists such as adenosine diphosphate, but not by strong agonists like thrombin [41],[42]. Propofol, on the contrary, inhibits platelet aggregation mediated by thrombin [43] that regulates also the expression of p-selectin on platelets.

## Conclusions

The marked and significant increase in pro-coagulant factors and consequent reduction in haemostatic system inhibitors we observed in the early post operative period suggests that a peri-operative thromboprophylaxis may be beneficial in cancer patients undergoing laparoscopic radical prostatectomy especially when a robot-assistance is used.

## Abbreviations

LRP: Laparoscopic prostatectomy

RALP: Robot-assisted laparoscopic prostatectomy

TIVA-TCI: Total intravenous anesthesia with target-controlled infusion

BAL: Balanced inhalation anaesthesia

TED: Thromboembolic disease

F1?+?2: Prothrombin fragment 1?+?2

TAT: Thrombin-antithrombin complexes

PAI-1: Plasminogen activator inhibitor type 1

FVIII: Factor VIII

AT: Antithrombin

PC: Protein C

PS: Protein S

## Competing interests

Sofra M, Antenucci A, Gallucci M, Mandoj C, Papalia R, Claroni C, Monteferrante I, Torregiani G, Gianaroli V, Sperduti I and Forastiere E: No interest declared.

## Authors¿ contributions

MS and EF contributed to conception and design of the study, acquisition, analysis and interpretation of data. AA, MG, CM and IS worked on the acquisition, analysis and interpretation of data. RP, CC, IM, GT and VG contributed to acquisition of data. All Authors were involved in drafting the manuscript or revising it critically for important intellectual content and gave final approval of the version to be published.
